# Associations of dietary intakes of calcium, magnesium and soy isoflavones with osteoporotic fracture risk in postmenopausal women: a prospective study

**DOI:** 10.1017/jns.2022.52

**Published:** 2022-08-01

**Authors:** Yong Cui, Hui Cai, Yutang Gao, Qi Dai, Gong Yang, Wei Zheng, Xiao-Ou Shu

**Affiliations:** 1 Division of Epidemiology, Department of Medicine, Vanderbilt-Ingram Cancer Center, Vanderbilt Epidemiology Center, Vanderbilt University Medical Center, Nashville, TN, USA; 2 Department of Epidemiology, Shanghai Cancer Institute, Renji Hospital, Shanghai Jiaotong University School of Medicine, Shanghai, China

**Keywords:** Bone fractures, Calcium, Magnesium, Osteoporotic fractures, Postmenopausal women, Soy isoflavones, BMI, body mass index, Ca, calcium, CI, confidence interval, FFQ, food frequency questionnaire, HR, hazard ratios, Mg, magnesium, non-OF, non-osteoporotic fractures, OF, osteoporotic fractures, SWHS, Shanghai Women's Health Study

## Abstract

The role of dietary factors in osteoporotic fractures (OFs) in women is not fully elucidated. We investigated the associations between incidence of OF and dietary calcium, magnesium and soy isoflavone intake in a longitudinal study of 48 584 postmenopausal women. Multivariable Cox regression was applied to derive hazard ratios (HRs) and 95 % confidence intervals (CIs) to evaluate associations between dietary intake, based on the averages of two assessments that took place with a median interval of 2⋅4 years, and fracture risk. The average age of study participants is 61⋅4 years (range 43⋅3–76⋅7 years) at study entry. During a median follow-up of 10⋅1 years, 4⋅3 % participants experienced OF. Compared with daily calcium intake ≤400 mg/d, higher calcium intake (>400 mg/d) was significantly associated with about a 40–50 % reduction of OF risk among women with a calcium/magnesium (Ca/Mg) intake ratio ≥1⋅7. Among women with prior fracture history, high soy isoflavone intake was associated with reduced OF risk; the HR was 0⋅72 (95 % CI 0⋅55, 0⋅93) for the highest (>42⋅0 mg/d) *v.* lowest (<18⋅7 mg/d) quartile intake. This inverse association was more evident among recently menopausal women (<10 years). No significant association between magnesium intake and OF risk was observed. Our findings provide novel information suggesting that the association of OF risk with dietary calcium intake was modified by Ca/Mg ratio, and soy isoflavone intake was modified by history of fractures and time since menopause. Our findings, if confirmed, can help to guide further dietary intervention strategies for OF prevention.

## Introduction

Osteoporosis is characterised by reduced bone mass and fragmentation of bone architecture, with a consequent increase in bone fragility and susceptibility to fractures^(1,2)^. Osteoporotic fractures (OFs) are a major cause of disability and reduced quality of life, particularly in postmenopausal women^(2)^. It has been estimated that approximately 1 in 2 women aged 50 years or older will experience an OF in their remaining lifetime, imposing a considerable economic burden on health services^(3–5)^. Previous studies have investigated relationships between OF and several nutritional and/or dietary factors; however, the results are mixed^(6,7)^. More information is needed on the roles of these modifiable factors in OF for the development of non-pharmacologic preventive strategies, accordingly.

Both calcium and magnesium are major components of bone and essential micronutrients required to maintain bone homeostasis and play important roles in bone health^(8,9)^. Dietary calcium intake has been recommended for osteoporosis prevention; however, the recommendations vary by country^(10,11)^. Furthermore, studies have shown that magnesium may interact with calcium and/or vitamin D, interfere with calciotropic hormones, and has been known as a natural calcium antagonist^(12–14)^. To date, information on the relationship between dietary magnesium intake and OF risk is sparse, and whether dietary magnesium intake is related to the risk of OF or may influence the estimation of the association between dietary calcium intake and OF is largely unknown.

Estrogen plays an important role in skeletal homeostasis and regulates bone remodelling^(15,16)^, while the decline in estrogen levels associated with menopause causes bone loss in women^(17)^. Estrogen exerts its effect on target cells through binding estrogen receptors (ERs);^(18)^ thus, modulators of ERs play an important role in bone health. Isoflavones, rich in soybeans and soy-based products, are major types of phytoestrogen, with a noticeable property as a natural selective ER modulator^(19)^. *In vitro* experiments and *in vivo* animal studies have shown that isoflavones have potential bone-specific effects via estrogenic/antiestrogenic effects and other biologic mechanisms^(20,21)^. Epidemiological and clinical evidence support that dietary isoflavones may attenuate menopause-induced osteoporotic bone loss^(22–24)^. However, information is still limited regarding whether the association between dietary isoflavone intake and OF risk is modified by timing regarding menopause and other host factors as previously suggested^(25)^.

In the present study, we investigated the incidence of OF and evaluated their associations with dietary calcium, magnesium and soy isoflavone intake in a prospective observational cohort of over 48 000 postmenopausal women.

## Methods

### Study population

**Fig. 1.**
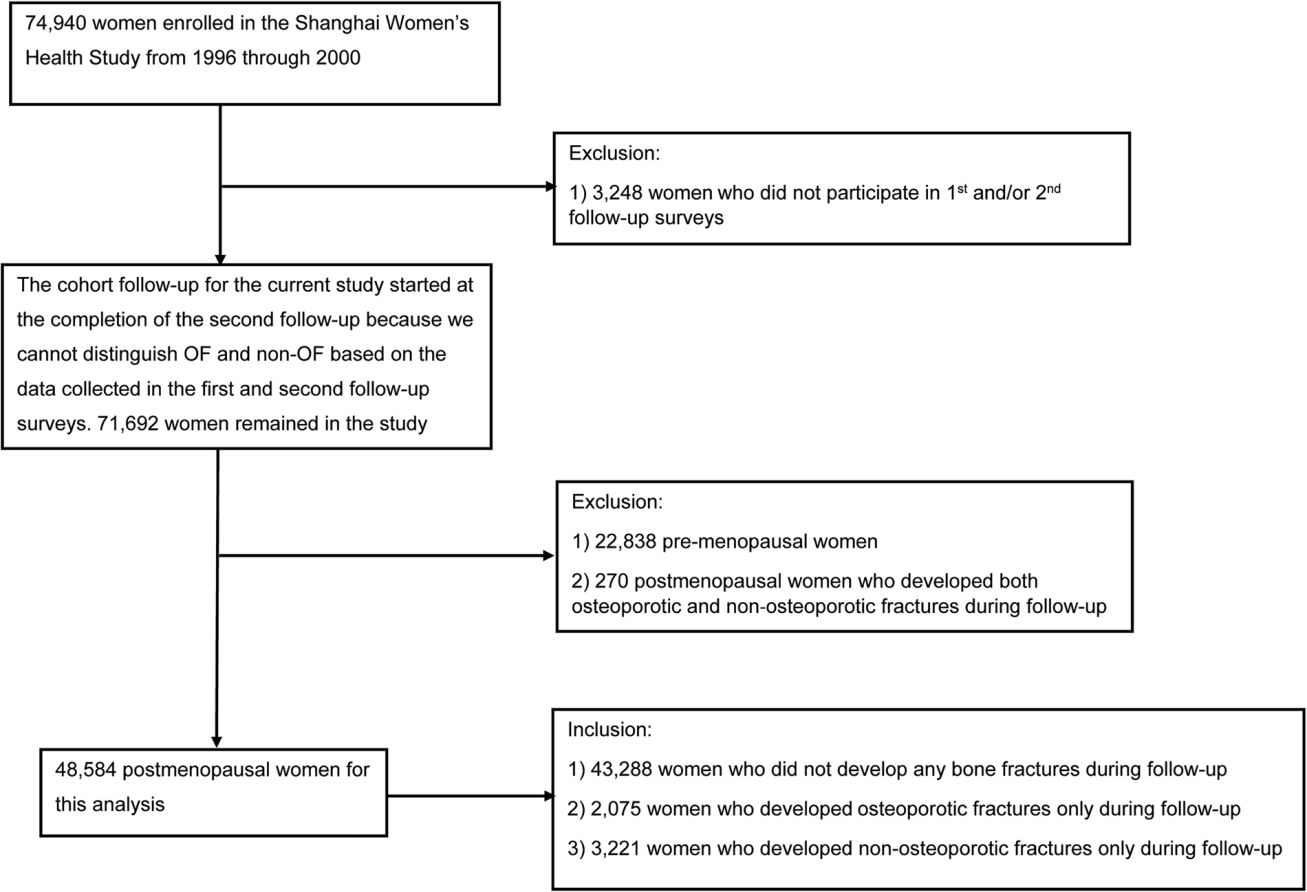
Flowchart for the participant selection process.

Participants of the study were drawn from the Shanghai Women's Health Study (SWHS), a large population-based prospective cohort study conducted in urban Shanghai, China. Detailed descriptions of the study design and methods have been published elsewhere^(26)^. Briefly, 74 940 women aged 40–74 years were recruited from seven typical urban communities in Shanghai between 1996 and 2000, with a 92 % response rate. At the study enrolment, each participant signed a consent form and completed an in-person survey conducted by trained interviewers. The information collected at enrolment included socio-demographic characteristics, dietary habits including soy intake, physical activity, menstrual and reproductive history, smoking, drinking and other lifestyle factors and exposures, as well as medical history. Anthropometric measurements were also taken. The cohort has been followed up between 2000 and 2016, through in-person surveys every 2–4 years, a total of five times to update exposure information and collect information on changes of health status, including bone fractures and vital status. Annual record linkages with the vital statistics registry were carried out to ensure a complete ascertainment of mortality information. Response rates for the five in-person follow-up surveys were over 91 % (99⋅7, 98⋅8, 95⋅0, 92⋅5 and 91⋅0 %, respectively). In the first and second follow-up surveys, participants were only asked about time (month and year) and anatomic site(s) of a bone fracture, but no information was collected on the cause of the fracture; thus, the type of bone fracture could not be assessed. Therefore, for the current analysis, we only included OF and non-osteoporotic fractures (non-OFs) that occurred after the second follow-up survey as outcomes of the study. In other words, the cohort follow-up for the current analysis started at the completion of the second follow-up. Fractures developed between the SWHS enrolment and the second follow-up survey were treated as history of bone fractures and the menopausal status was based on the data of the second follow-up survey. We excluded 3248 women who did not participate in the first and/or second follow-up surveys, 22 778 women with premenopausal status at the second follow-up survey, and 270 postmenopausal women who developed both OF and non-OF after the second follow-up survey, leaving a total of 48 584 postmenopausal women for the current analysis (Fig. 1). The SWHS was approved by the institutional review boards of all participating institutions. This study was conducted according to the guidelines laid down in the Declaration of Helsinki, and all procedures involving human subjects/patients were approved by the institutional review boards of all participating institutions. Written informed consent was obtained from all subjects/patients.

### Study variables and covariates assessment

Dietary information was collected using a validated food frequency questionnaire (FFQ)^(27)^ at the study enrolment and at the first FFQ follow-up for the present study. A total of eighty-one food items were included in the SWHS FFQ. For each food item or food group, subjects were asked how frequently (daily, weekly, monthly, annually or never) they consumed the food or food group, which was followed by a question on the amount consumed in lians per unit of time. Lian is a unit of weight in China (1 lian = 50 g). Soy intake assessed in the study included consumption of tofu, soy milk, fresh soybeans and other soy products. Daily intakes of calories, macro- and micronutrients, vitamin D, calcium, magnesium, protein, soy isoflavones and major isoflavone components (genistein, daidzein and glycitein) were derived from FFQ data by summing the products of individual food intake amounts and nutrient contents of food items based on the Chinese Food Composition Tables^(27,28)^. To improve the dietary assessment, we averaged the dietary intake data collected at study enrolment and the first follow-up and applied them in the present study. For those women (*n* = 2217, 4⋅5 %) who missed information on the dietary assessment at the first FFQ follow-up and/or were diagnosed with cancer, diabetes, stroke or myocardial infarction between the study enrolment and the first follow-up survey, only dietary intake data at enrolment were used due to the concern that these diseases and associated treatments would change dietary habits. Dietary intakes were categorised by quartile distributions for the analyses. Calcium and magnesium intakes were categorised from ≤400 to >800 mg/d and from ≤200 to >400 mg/d, respectively, while soy isoflavone intake was categorised by quartile distributions.

### Outcomes

The primary outcome of interest was the occurrence of OF. During the in-person follow-up surveys, participants were asked if they had a bone fracture since the last survey. If a participant answered ‘yes’, she was asked to provide further information on time (month and year), anatomic site(s) and cause of fracture. Fracture sites were coded with ICD-9 or ICD-10. Fractures in anatomic sites commonly associated with osteoporosis include ICD-9 codes 805, 806, 807, 808, 810, 812, 813, 818, 819, 820, 821, 822, 823, 824 and ICD-10 codes S22, S32, S42, S52, S72, S82 and M80. For causes of fractures, participants could select (1) car accident or physical trauma, (2) fall when riding a bicycle, (3) fall by sliding/fall from standing height, (4) fall down from a high place (providing height in m), and (5) others (specify the cause). OFs were defined as low-trauma bone fractures (e.g. due to falls by sliding/from standing height) and occurring in anatomic sites commonly associated with osteoporosis, with exception of those caused by malignancy or other pathologic fractures not related osteoporosis^(25,29)^. Non-OFs included any fracture other than OF. Because trauma, which is unrelated to diet, is the major cause of non-OF, we also included non-OF as a comparison group to evaluate the validity of the outcome assessment and study findings^(30,31)^.

### Statistical analysis

Included in the current analysis were 48 584 postmenopausal women. We used the Kaplan–Meier product-limit method to estimate the 10-year occurrence rate, and a log-rank test to compare 10-year occurrence rates among participant groups defined by covariates. Multivariable Cox regression model was applied to evaluate the associations of dietary calcium intake, dietary magnesium intake and soy isoflavone intake with incidence of OF and non-OF, measured by hazard ratios (HRs) and 95 % confidence intervals (CIs). Entry time was the date of the second follow-up survey completion, and exit time was the date of fracture occurrence, date of cancer, stroke or myocardial infarction diagnosis (due to a concern that these events and their associated treatments may change a participant's dietary habits), date at death, or last follow-up, whichever came first. Covariates adjusted for in the models included known/suspected risk factors for bone fractures, based on literature and factors that were significantly associated with bone fracture risk in the univariate analysis of our own data. These included age (continuous variable), educational level (less than high school/high school graduate/higher than high school), cigarette smoking status (never/ever), alcohol consumption (yes/no), regular exercise (yes/no), body mass index (BMI, continuous variable), calcium supplement use (non-user/ever-user), comorbidity (Charlson's score, 0/1/≥2), fracture history (yes/no) and dietary intakes of calories and vitamin D (quartiles). All covariates were based on information collected at study entry except age, which was collected at the second follow-up survey completion, and dietary intakes of calories, calcium, magnesium and vitamin D, which were the averages of baseline and first FFQ follow-up survey data. Dietary calcium and magnesium intakes were mutually adjusted in the model. The same models were applied in multiplicative interactions between isoflavone/each composition and bone fracture history or year(s) since menopause. Proportional hazard assumption was assessed by adding an interaction term between an exposure and time (exposure*log [time]) in the model for testing. No major violation was discovered for the proportionality assumption. All statistical tests were based on two-tailed probability and a significance level set at alpha (*α*) < 0⋅05.

## Results

During a median follow-up of 10⋅1 years (interquartile range: 9⋅3–11⋅0) since the completion of the second follow-up survey, among 48 584 postmenopausal women, 2075 developed OF, 3221 developed non-OF and 43 288 remained free of any fractures. Table 1 shows the 10-year occurrence of two types of bone fractures by participant characteristics among postmenopausal women. For OF, a higher 10-year occurrence rate was observed among women with a prior history of fractures, older age (especially older than 69 years), low income, ever cigarette smoking, BMI less than 18⋅5, regular exercise, comorbidity (especially Chalson's comorbidity score ≥2), longer breast-feeding (especially >36 months) and ever use of calcium supplements. For non-OF, however, the rate did not increase with age. A higher 10-year occurrence rate was seen among women with a prior history of fractures, high educational level, normal BMI (18⋅5–25⋅0), regular exercise, breast-feeding for 1–36 months and ever use of calcium supplements.

**Table 1.** 10-year occurrence rate by participant characteristics and fracture type among postmenopausal women, the SWHS (*N* = 48 584)

Variables	Osteoporotic fracture			Non-fracture		
	Event/All	10-year occurrence (%)	*P*-value	Event/All	10-year occurrence (%)	*P*-value
History of any bone fractures						
No	1324/36 613	3⋅86		2104/36 613	6⋅02	
Yes	751/11 971	7⋅12	<0⋅001	1117/11 971	10⋅24	<0⋅001
Age (at baseline, years)						
≤49	72/2467	3⋅01		172/2467	6⋅86	
50–59	614/18 556	3⋅44		1292/18 556	7⋅07	
60–69	738/16 018	5⋅04		1091/16 018	7⋅32	
>69	651/11 543	6⋅47	<0⋅001	666/11 543	6⋅57	0⋅27
Household income						
Low	373/8230	5⋅01		508/8230	6⋅73	
Middle	1553/36 237	4⋅65		2445/36 237	7⋅13	
High	149/4117	3⋅79	0⋅01	268/4117	6⋅74	0⋅39
Educational level						
<High school	1331/30 078	4⋅79		1896/30 078	6⋅70	
High school	446/11 488	4⋅21		816/11 488	7⋅45	
>High school	298/7018	4⋅67	0⋅06	509/7018	7⋅75	0⋅003
Smoking status						
Non-smoker	1996/46 962	4⋅60		3129/46 962	7⋅05	
Ever smoker	79/1622	5⋅86	0⋅03	92/1622	6⋅41	0⋅38
Alcohol consumption						
Never	2021/47 473	4⋅62		3136/47 473	7⋅00	
Ever	54/1111	5⋅53	0⋅16	85/1111	8⋅30	0⋅12
BMI						
<18⋅5	79/1587	5⋅73		99/1587	6⋅83	
18⋅5–25	1152/27 504	4⋅53		1922/27 504	7⋅37	
>25	844/19 493	4⋅69	0⋅12	1200/19 493	6⋅57	0⋅009
Regular exercise						
No	1130/27 890	4⋅34		1801/27 890	6⋅80	
Yes	945/20 694	5⋅04	0⋅009	1420/20 694	7⋅35	0⋅02
Charlson's Comorbidity Score						
0	1626/40 117	4⋅36		2656/40 117	6⋅92	
1	152/3080	5⋅58		215/3080	7⋅75	
≥2	297/5387	6⋅24	<0⋅001	350/5387	7⋅44	0⋅11
Breast-feeding (Months)						
0	326/7883	4⋅56		498/7883	6⋅76	
1–14	630/17 595	3⋅78		1256/17 595	7⋅27	
14⋅1–36	732/15 852	5⋅06		1072/15 852	7⋅32	
>36	387/7254	5⋅92	<0⋅001	395/7254	6⋅07	0⋅006
Calcium supplement use						
Never	1150/28 334	4⋅39		1755/28 334	6⋅55	
Ever	925/20 250	4⋅98	0⋅004	1466/20 250	7⋅71	<0⋅001

Table 2 shows the associations of dietary calcium and magnesium intakes with OF overall and by calcium/magnesium (Ca/Mg) intake ratio and calcium supplement use. Overall, no significant association was found between dietary calcium intake and OF. Analysis stratified by the dietary Ca/Mg ratio median (ratio <1⋅7 or ≥1⋅7, median cut), however, showed that, compared with lower calcium intake (≤400 mg/d), higher calcium intake (>400 mg/d) was associated with about a 40–50 % reduction of OF risk among women with a dietary Ca/Mg ratio ≥1⋅7 (HR 0⋅63, 95 % CI 0⋅48, 0⋅82 to HR 0⋅52, 95 % CI 0⋅34, 0⋅80). This association was observed among women with (range from HR 0⋅67, 95 % CI 0⋅44, 1⋅01 to HR 0⋅51, 95 % CI 0⋅28, 0⋅94) and without (range from HR 0⋅65, 95 % CI 0⋅41, 1⋅02 to HR 0⋅53, 95 % CI 0⋅29, 0⋅99) use of calcium supplements. However, no significant association was observed between dietary calcium intake and OF among women with a dietary Ca/Mg ratio <1⋅7. No significant associations were observed between dietary magnesium intake and OF or non-OF overall or by Ca/Mg ratio and calcium supplement use. Our observations showed that the associations of calcium or magnesium intakes did not vary by history of bone fractures and no significant association was found between calcium intake and non-OF, regardless of Ca/Mg ratio and calcium supplement use (data not shown).

**Table 2.** Associations of calcium and magnesium intakes with osteoporotic fracture by Ca/Mg ratio and Ca supplement use among postmenopausal women

All women	Overall	Ca/Mg ratio < median (1⋅7)^a^	Ca/Mg ratio ≥ median (1⋅7)^a^
	Adjusted HR (95 % CI)^b^	Adjusted HR (95 % CI)^b^	Adjusted HR (95 % CI)^b^
Calcium intake (mg/d)			
≤400	1⋅00 (ref.)	100 (ref)	1⋅00 (ref.)
401–600	0⋅99 (0⋅88–1⋅12)	1⋅06 (0⋅89–1⋅27)	0⋅63 (0⋅48–0⋅82)
601–800	0⋅97 (0⋅80–1⋅17)	1⋅18 (0⋅72–1⋅92)	0⋅63 (0⋅46–0⋅87)
>800	0⋅90 (0⋅67–1⋅21)	1⋅98 (0⋅68–5⋅81)	0⋅52 (0⋅34–0⋅80)
Magnesium intake (mg/d)			
≤200	1⋅00 (ref.)	1⋅00 (ref.)	1⋅00 (ref.)
201–300	0⋅95 (0⋅82–1⋅10)	0⋅96 (0⋅79–1⋅17)	1⋅13 (0⋅85–1⋅51)
301–400	0⋅93 (0⋅75–1⋅17)	0⋅96 (0⋅70–1⋅31)	1⋅08 (0⋅75–1⋅55)
400	1⋅01 (0⋅72–1⋅41)	0⋅62 (0⋅32–1⋅19)	1⋅43 (0⋅88–2⋅33)
Calcium supplement non-users			
Calcium intake (mg/d)			
≤400	1⋅00 (ref.)	1⋅00 (ref.)	1⋅00 (ref.)
401–600	0⋅97 (0⋅83–1⋅14)	1⋅03 (0⋅81–1⋅29)	0⋅59 (0⋅41–0⋅84)
601–800	0⋅95 (0⋅73–1⋅23)	0⋅92 (0⋅47–1⋅80)	0⋅65 (0⋅41–1⋅02)
>800	0⋅90 (0⋅59–1⋅39)	1⋅75 (0⋅50–6⋅14)	0⋅53 (0⋅29–0⋅99)
Magnesium intake (mg/d)			
≤200	1⋅00 (ref.)	1⋅00 (ref.)	1⋅00 (ref.)
201–300	0⋅91 (0⋅75–1⋅11)	0⋅88 (0⋅70–1⋅12)	1⋅11 (0⋅75–1⋅63)
301–400	0⋅88 (0⋅65–1⋅18)	0⋅84 (0⋅57–1⋅24)	0⋅99 (0⋅59–1⋅65)
>400	0⋅87 (0⋅55–1⋅39)	0⋅64 (0⋅28–1⋅47)	1⋅18 (0⋅58–2⋅40)
Calcium supplement users			
Calcium intake (mg/d)			
≤400	1⋅00 (ref.)	1⋅00 (ref.)	1⋅00 (ref.)
401–600	1⋅03 (0⋅85–1⋅25)	1⋅13 (0⋅84–1⋅52)	0⋅67 (0⋅44–1⋅01)
601–800	0⋅99 (0⋅75–1⋅30)	1⋅65 (0⋅80–3⋅38)	0⋅63 (0⋅39–1⋅02)
>800	0⋅89 (0⋅58–1⋅36)	2⋅05 (0⋅23–18⋅5)	0⋅51 (0⋅28–0⋅94)
Magnesium intake (mg/d)			
≤200	1⋅00 (ref.)	1⋅00 (ref.)	1⋅00 (ref.)
201–300	1⋅01 (0⋅79–1⋅30)	1⋅12 (0⋅80–1⋅57)	1⋅14 (0⋅74–1⋅74)
301–400	1⋅03 (0⋅73–1⋅46)	1⋅18 (0⋅70–1⋅98)	1⋅13 (0⋅67–1⋅91)
>400	1⋅22 (0⋅74–2⋅00)	0⋅59 (0⋅20–1⋅71)	1⋅63 (0⋅83–3⋅22)

a In the group with Ca/Mg ratio < median (1⋅7), mean Ca/Mg ratio = 1⋅44; in the group with Ca/Mg ratio ≥ median (1⋅7), mean Ca/Mg ratio = 2⋅07.

b Adjusting for: Age, history of bone fracture, income, educational level, cigarette smoking status, alcohol consumption, regular exercise, BMI, Charlson's Score, breasting time, calcium supplement use, daily dietary intake of calories and vitamin D. Dietary calcium and magnesium intakes were mutually adjusted in the model.

Table 3 shows the associations of soy isoflavone intake with OF overall and by prior fracture history among postmenopausal women. Overall, no associations were observed between soy isoflavone intake and OF; however, a significant interaction between soy isoflavone intake and prior fracture history was detected (*P* for interaction = 0⋅002). Among women with a prior bone fracture history, compared with the lowest quartile (<1⋅7 mg/d), the highest quartile (>42⋅0 mg/d) of soy isoflavone intake was associated with a 28 % reduction of OF risk (HR 0⋅72, 95 % CI 0⋅55, 0⋅93). By contrast, high isoflavone intake was associated with an elevated OF risk, seen among women without a prior fracture history (HR 1⋅22, 95 % CI 1⋅01, 1⋅48). Similar association patterns were also observed between major components of soy isoflavones (daidzein, genistein and glycitein) and OF. No significant association was observed between soy isoflavone intake and non-OF, regardless of prior history of bone fractures (data not shown).

**Table 3.** Associations of soy isoflavones intake with osteoporotic fracture by any bone fracture history among postmenopausal women

	Adj. HR (95 % CI)^a^			*P* for interaction^b^
	Overall	With BF history	Without BF history	
Soy isoflavone intake (mg/d)				
<18⋅7	1⋅00 (ref.)	1⋅00 (ref.)	1⋅00 (ref)	
18⋅7–29⋅0	1⋅08 (0⋅95–1⋅22)	1⋅07 (0⋅87–1⋅31)	1⋅08 (0⋅92–1⋅27)	0⋅002
29⋅1–42⋅0	1⋅02 (0⋅89–1⋅16)	0⋅84 (0⋅67–1⋅05)	1⋅13 (0⋅95–.34)	
>42⋅0	1⋅01 (0⋅86–1⋅18)	0⋅72 (0⋅55–0⋅93)	1⋅22 (1⋅01–1⋅48)	
Daidzein intake (mg/d)				
<7⋅7	1⋅00 (ref.)	1⋅00 (ref.)	1⋅00 (ref.)	
7⋅7–12⋅0	1⋅06 (0⋅93–1⋅20)	1⋅05 (0⋅86–1⋅29)	1⋅07 (0⋅91–1⋅25)	0⋅004
12⋅17⋅6	1⋅00 (0⋅87–1⋅14)	0⋅83 (0⋅66–1⋅04)	1⋅11 (0⋅93–1⋅31)	
>17⋅6	1⋅02 (0⋅88–1⋅19)	0⋅75 (0⋅58–0⋅97)	1⋅21 (1⋅00–1⋅47)	
Genistein intake (mg/d)				
<10⋅6	1⋅00 (ref.)	1⋅00 (ref.)	1⋅00 (ref.)	
10⋅6–16⋅6	1⋅05 (0⋅92–1⋅19)	1⋅03 (0⋅84–1⋅27)	1⋅06 (0⋅90–1⋅24)	0⋅004
16⋅7–24⋅2	1⋅00 (0⋅88–1⋅15)	0⋅83 (0⋅67–1⋅04)	1⋅11 (0⋅94–1⋅31)	
>24⋅2	1⋅03 (0⋅89–1⋅20)	0⋅76 (0⋅59–0⋅98)	1⋅22 (1⋅01–1⋅48)	
Glycitein intake (mg/d)				
<1⋅6	1⋅00 (ref.)	1⋅00 (ref.)	1⋅00 (ref.)	
1⋅6–2⋅4	1⋅11 (0⋅98–1⋅25)	1⋅05 (0⋅86–1⋅29)	1⋅14 (0⋅97–1⋅34)	0⋅02
2⋅5–3⋅4	1⋅03 (0⋅90–1⋅19)	0⋅89 (0⋅71–1⋅12)	1⋅12 (0⋅95–1⋅34)	
>3⋅4	1⋅03 (0⋅87–1⋅21)	0⋅77 (0⋅59–1⋅01)	1⋅21 (0⋅99–1⋅49)	

a Adjusting for: Age, BF history, income, educational level, cigarette smoking status, alcohol consumption, regular exercise, BMI, Charlson's Score, breasting time, calcium supplement use, daily dietary intake of calories, vitamin D, calcium and magnesium.

b *P*-value for interaction between soy isoflavones and BF history.

We further examined associations of soy isoflavone intake with bone fractures by year(s) since menopause among postmenopausal women (Table 4). For women with a prior fracture history, soy isoflavone intake was found to be inversely associated with OF risk among women who became menopausal within 10 years (HR 0⋅80, 95 % CI 0⋅56, 1⋅16 for isoflavone intake between 18⋅7 and 29⋅0 mg/d, HR 0⋅65, 95 % CI 0⋅43, 1⋅00 for isoflavone intake between 29⋅1 and 42⋅0 mg/d, HR 0⋅59, 95 % CI 0⋅37, 0⋅96 for isoflavone intake >42⋅0 mg/d), but not among those who had been menopausal for over 10 years. No association was observed between soy isoflavone intake and non-OF risk among postmenopausal women, regardless of prior history of bone fracture and year(s) since menopause (data not shown).

**Table 4.** Associations of soy isoflavones intake with osteoporotic fractures by year(s) since menopause among postmenopausal women

Year(s) since menopause	0–10 years		>10 years		*P* for interaction^b^
	Event/participant	Adj. HR (95 %CI)^a^	Event/participant	Adj. HR (95 %CI)^a^	
Women with BF history					
Soy isoflavone Intake (mg/d)					
<18⋅7	65/942	1⋅00 (ref.)	128/1736	1⋅00 (ref)	
18⋅7–29⋅0	59/1039	0⋅80 (0⋅56–1⋅16)	149/1641	1⋅21 (0⋅94–1⋅54)	0⋅12
29⋅1–42⋅0	47/993	0⋅65 (0⋅43–1⋅00)	132/1723	0⋅94 (0⋅72–1⋅23)	
>42⋅0	44/956	0⋅59 (0⋅37–0⋅96)	127/1824	0⋅77 (0⋅56–1⋅06)	
Women without BF history					
Soy isoflavone intake (mg/d)					
<18⋅7	124/4130	1⋅00 (ref.)	183/4557	1⋅00 (ref.)	
18⋅7–29⋅0	129/4399	1⋅01 (0⋅78–1⋅30)	193/4222	1⋅14 (0⋅92–1⋅40)	0⋅74
29⋅1–42⋅0	136/4308	1⋅09 (0⋅83–1⋅43)	201/4337	1⋅15 (0⋅93–1⋅44)	
>42⋅0	136/3979	1⋅24 (0⋅91–1⋅70)	222/4577	1⋅21 (0⋅94–1⋅56)	

a Adjusting for: Age, income, educational level, cigarette smoking status, alcohol consumption, regular exercise, BMI, Charlson's Score, breasting time, calcium supplement use, daily dietary intake of calories, vitamin D, calcium and magnesium.

b *P*-value for interaction between soy isoflavones and year(s) since menopause.

## Discussion

In this large-scale longitudinal study, we found that dietary calcium intake was inversely associated with OF among postmenopausal women who had a dietary Ca/Mg ratio above 1⋅7, regardless of calcium supplement use. We also found that high levels of soy isoflavone intake and intakes of major isoflavone components (genistein, daidzein or glycitein) were associated with a reduced risk of OF in postmenopausal women who had a prior history of bone fracture, particularly in those who became menopausal within 10 years of dietary assessments.

Although dietary calcium intake has been recommended for osteoporosis prevention, previous studies examining the associations of dietary calcium intake with OF risk have yielded mixed results^(10,32–36)^. In an early systematic review and meta-analysis^(32)^, Cumming *et al.* analysed fourteen studies of calcium supplements (including four randomised trials), eighteen studies of dietary calcium and hip fracture (no randomised trials), five studies of dietary calcium and other fracture sites (no randomised trials), as well as sixteen additional observational studies of dietary calcium and hip fracture (eleven case-control studies and five cohort studies). Meta-analysis of sixteen observational studies on dietary calcium and hip fracture revealed a significant heterogeneity across studies. The pooled estimate on dietary calcium intake and hip structure was 0⋅96 (95 % CI 0⋅93, 0⋅99) per 300 mg/d increase in calcium intake. The authors concluded that the overall result supports the current clinical and public health policy of recommending increased calcium intake among older women for fracture prevention. A recent systematic review and meta-analysis^(10)^ showed that vitamin D supplementation alone was associated with a reduced risk of fractures, and daily use of both calcium and vitamin D supplementation reduced the risk of hip fracture. However, several other studies found no strong evidence that dietary calcium intake was associated with reduced risk of OFs^(33–36)^. For example, using data from the National Osteoporosis Risk Assessment (NORA) study^(36)^, Nieves *et al.* reported that calcium and vitamin D intakes influenced bone mass but not short-term fracture risk in Caucasian postmenopausal women. In the present study, we found that among postmenopausal women with a dietary Ca/Mg ratio >1⋅7, among whom the mean Ca/Mg intake ratio was 2⋅07, dietary calcium intake was inversely associated with OF. However, no significant association was found among women whose Ca/Mg intake ratio was ≤1⋅7, among whom the mean Ca/Mg intake ratio was 1⋅44. Ca/Mg ratio has been previously suggested to modify the effect of Ca on mortality or cancer incidence. Dai and his colleagues reported that among men with a Ca/Mg ratio >1⋅7, increased intake of Ca was associated with reduced risk of total mortality and mortality due to cancer^(13)^. In a recent study, it was also reported that the inverse association between calcium intake and distal colorectal cancer was modified by the Ca/Mg ratio^(37)^. Our results are in line with findings from these previous observational studies supporting that the Ca effect on health may be modified by Ca/Mg ratio. Additionally, a recent precision-based randomised trial showed that optimal magnesium status was important for optimising vitamin D status, which is essential to calcium absorption^(38,39)^. Our study finding suggests that a proper Ca/Mg ratio may be required for bone health effects of calcium, and failure to take this property into consideration when evaluating the health effects of calcium may lead to erroneous conclusions.

There were several reports on dietary magnesium intake and risk of osteoporosis fractures; however, findings were conflicting. In the Women's Health Initiative Observational Study, Orchard *et al.* reported that a lower magnesium intake is associated with lower bone mineral density (BMD) of the hip, but a magnesium intake slightly greater than the Recommended Dietary Allowance is associated with increased lower-arm and wrist fractures^(40)^. A systematic review revealed a positive correlation between magnesium intake and BMD, but no association was found between high magnesium intake and fracture^(41)^. Consistently, we also did not find a significant association between magnesium intake and OF or non-OF. Interestingly, a recent study investigated both magnesium intake and serum level of magnesium, and found an inverse association between magnesium concentration in serum and fracture risk in middle-aged Caucasian men, but a null association for magnesium intake^(42)^. Additional research is needed to fully understand the role of magnesium intake and absorption in fracture.

Studies have well documented that a prior history of fractures is an important risk factor for osteoporosis and OF^(43,44)^. For example, Toth *et al.*^(43)^ evaluated a retrospective, observational study of a total of 35 146 women aged 55–90 years. The authors found that among 7180 hip, 2786 clinical vertebral, and 25 180 non-hip/non-vertebral (NHNV) index fractures, 38 % of women with hip, 38 % with clinical vertebral and 25 % with NHNV index fractures had one or more previous fractures. Following any index fracture, cumulative incidence of a new fracture was over 11 % over the subsequent 24 months. These data demonstrate that almost one-third of women aged 55–90 years suffering a new fracture have had a previous fragility fracture. Among postmenopausal women from the SWHS who had a prior history of bone fracture, 7⋅1 % developed OF during the 10-year follow-up, while among those without a history of bone fracture, the 10-year occurrence rate was 3⋅9 %. Furthermore, we found that among women with a prior history of fractures, a higher level of soy isoflavone intake (>42 mg/d) was associated with a 28 % reduction of OF risk. Similar association patterns were also observed between major components of soy isoflavones (genistein, daidzein and glycitein) and OF. Thus, our finding supports the notion that prior history of fractures is an important risk factor for OF, and further provides the first evidence that prior history of fractures modifies the association of soy isoflavone intake with OF in postmenopausal women. On the other hand, we found that high dietary isoflavone intake was associated with an increased risk of OF among women without a bone fracture history.

Menopause is associated with increased bone resorption and decreased BMD because estrogen levels drop. Isoflavone has both estrogenic/antiestrogenic effects^(20,21)^. Several studies have reported that soy isoflavones attenuate menopause-induced osteoporotic bone loss and fractures among women^(22–25)^. A review of randomised controlled trials suggested that soy isoflavone consumption during menopausal transition may prevent a reduction in BMD and promote bone health^(24)^. Indeed, our previous study found that soy isoflavone intake was associated with a reduced risk of incident fractures in pre-/perimenopausal breast cancer survivors^(25)^. Findings of the present study support the notion that soy isoflavone intake may protect against menopause-related osteoporosis and OF among high-risk women, i.e. those with a prior history of bone fractures. We have no ready explanation for the opposite association, i.e. an increase of OF with high isoflavone intake found for women without a prior history of bone fractures. We speculate that women without a history of bone fracture may have high levels of endogenous estrogen, and among them, isoflavones may exert their antiestrogenic effects giving dual estrogen agonist and antagonistic effects of isoflavones. This hypothesis will need to be evaluated in further studies.

The strengths of the present study include the prospective study design, large sample size, high response rates, repeated dietary assessments using a validated FFQ and parallel analyses of non-OF. However, our study also has several limitations. First, our information on fracture incidences and exposures was self-reported. No information was available on BMD and history of OF. Thus, misclassification on outcome assessment is possible, although a major bone fracture such as OF is more likely be accurate. The latter is supported by a validity study which compared self-reported fractures with data from medical records and found that the validity of self-reports for hip and forearm/wrist fractures was high^(45)^. In our study, we defined OF as low-trauma bone fractures (e.g. due to falls by sliding/fall from standing height) and occurring in anatomic sites commonly associated with osteoporosis, which takes into consideration both cause and anatomic site(s) to minimise the outcome misclassification bias. We found that OF and non-OF had different associations with age and several other socio-economic and lifestyle factors, in addition to the different association patterns with calcium and isoflavones. These data provide indirect support to the validity of our assessment of OF, as well as our study findings. Second, since our study was not originally designed for investigating OF, information on a number of potential risk factors for osteoporosis and OF, such as a family history of osteoporosis and hyperthyroidism or hyperparathyroidism, was not collected in the study. Thus, residual confounding may remain. Third, while we used data from two FFQs administered at the baseline survey and the first follow-up survey to improve the dietary assessment, changes in dietary intake during subsequent follow-ups were not captured, which may affect the associations between nutrients under study and fracture risk.

In summary, our study shows that among postmenopausal women, the calcium and OF association was modified by the Ca/Mg ratio. Dietary calcium intake more than 400 mg daily was associated with about a 40–50 % reduction of OF risk only when a daily dietary Ca/Mg ratio is equal to or above 1⋅7. Calcium intake was not associated with OF when Ca/Mg ratio was less than 1⋅7. We also provide evidence that a high level of soy isoflavone intake is associated with a reduced risk of OF, especially in postmenopausal women who had a prior bone fracture history and were within 10 years of menopause. These novel findings, if confirmed, would not only have a direct impact on the development strategies to prevent osteoporosis and OF among Chinese women, but also contribute to our understanding of the interplays among nutrients and between host characteristics and nutrient intake, as well as time of dietary intake on bone health, for postmenopausal women in general.

## Notes

Data will be provided upon request.

The SWHS was approved by the institutional review boards of all participating institutions.

All participants provided written informed consent.

All authors consent to the publication of this manuscript.

SWHS data used in this research project will be made available following the study data sharing policy posted online at: https://swhs-smhs.app.vumc.org/smhs_index.php.
